# Enhanced i‐Motif Stability through Consecutive 2′,2′‐Difluorocytidine Incorporation

**DOI:** 10.1002/chem.202503008

**Published:** 2025-11-19

**Authors:** Arnau Domínguez, Cristina Cabrero, Irene Gómez‐Pinto, Carme Fàbrega, Raimundo Gargallo, Ramon Eritja, Carlos González, Anna Aviñó

**Affiliations:** ^1^ Department of Surfactants and Nanobiotechnology Institute for Advanced Chemistry of Catalonia (IQAC‐CSIC) Jordi Girona 18–26 Barcelona 08034 Spain; ^2^ Biomedical Research Network Center for Bioengineering, Biomaterials and Nanomedicine (CIBER‐BBN) Instituto de Salud Carlos III Monforte de Lemos, 3–5 Madrid 28029 Spain; ^3^ Department of Inorganic and Organic Chemistry University of Barcelona (UB) Martí i Franquès, 1–11 Barcelona 08028 Spain; ^4^ Physical Biological Chemistry Blas Cabrera Institute of Physical Chemistry Serrano 119 Madrid 28006 Spain; ^5^ Department of Chemical Engineering and Analytical Chemistry University of Barcelona (UB) Martí i Franquès, 1–11 Barcelona 08028 Spain

**Keywords:** fluorine modifications, gemcitabine, I‐motif, NMR Structure, thermal stability

## Abstract

Chemical modifications of nucleic acids are widely used to tune stability and functionality in therapeutic and nanotechnological applications. Among these, fluorinated cytidine derivatives such as 2′‐fluoro‐arabinocytidine (2′F‐araC) and 2′‐fluoro‐ribocytidine (2′F‐riboC) have been shown to influence i‐motif structures differently, with 2′F‐araC strongly stabilizing and 2′F‐riboC exerting a mildly deleterious effect. In this study, we investigate the impact of gemcitabine (2′‐deoxy‐2′,2′‐difluorocytidine, dFdC) on i‐motif stability. dFdC exhibits small effects in single or double substituted sequences, but a pronounced stabilization when multiple consecutive residues are incorporated. Thermal and pH‐dependent analyses demonstrate that sequences containing fully substituted dFdC maintain i‐motif folding at neutral pH and show enhanced thermal stability. Structural insights suggest that this stabilization arises from a combination of factors, such as hyperconjugative interactions, hydrogen bonding, and dipole alignment, while the adaptable sugar conformation mitigates destabilizing minor groove contacts observed in other more rigid modifications, such as 2′‐F‐riboC. Cooperative interactions among adjacent dFdC residues and potential changes in hydration may play a key factor in reinforcing stability. These results highlight the unique capacity of dFdC to enhance i‐motif robustness and suggest that strategically placed difluoro substitutions can be exploited to design i‐motifs with improved stability, expanding their potential in biotechnology and therapeutic applications.

## Graphical abstract

Gemcitabine (dFdC) is a unique nucleoside containing two fluorine atoms at the 2′‐position. Using circular dichroism (CD) and nuclear magnetic resonance spectroscopy, we discovered that consecutive dFdC confer remarkable thermal and pH i‐motif stability. In particular, the molecular surface of the fully modified human vertebrate i‐motif structure shows the confluence of the fluorine atoms in green, which contributes to the overall stabilisation.

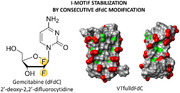

## 1 Introduction

DNA sequences can form secondary structures stabilized by nonWatson‐Crick base pairing. Among them, G‐quadruplex and i‐motifs are the most representative and have been identified at key genomic regions, including telomeres and oncogene promoters.^[^ ^1^, ^2^ ^]^ The biological implications of these structures in cellular processes and diseases have been subject of intense studies in recent years.^[^ ^3^, ^4^ ^]^

The i‐motif is a four‐stranded DNA structure formed by two intercalated duplexes stabilized by hemi‐protonated C─C^+^ base pairs. Since these interactions require cytosine protonation, folding of i‐motif‐forming oligonucleotides is more favorable under acidic conditions. Despite this pH‐sensitivity, the in vivo existence of i‐motifs and their detection have recently been demonstrated, suggesting a possible implication in biological processes.^[^ ^5^, ^6^, ^7^, ^8^ ^]^

Extensive work has examined the topology, stability, and potential therapeutic uses of i‐motif structures, including the search for specific ligands.^[^ ^9^, ^10^, ^11^ ^]^ In addition to their biological functions, i‐motif structures have received considerable attention due to their unique pH‐switching capacity. This property is particularly valuable for analytical and biomedical purposes,^[^ ^12^ ^]^ since pH is known to be a key regulator of multiple cellular functions.^[^ ^13^ ^]^ For instance, intracellular acidification is associated with apoptosis in many cancer cells, which exhibit a reversed pH gradient between intracellular and extracellular environments compared to normal cells.^[^ ^14^ ^]^

Numerous strategies have been developed to modulate acid‐base and thermal i‐motif stability. Of special interest are the incorporation of cytosine analogues, such as pseudoisocytidines^[^ ^15^ ^]^ and modifications in the loop regions, as well as the conjugation of molecules at the terminal positions of i‐motif forming sequences. Recent studies indicate that the stabilization of i‐motif at neutral pH may be achieved by end‐ligation^[^ ^16^ ^]^ or through the presence of an adjacent duplex structure.^[^ ^17^ ^]^ Although the hemi‐protonated base pair C─C^+^ is the primary stabilization factor, structural insights from NMR and X‐ray crystallographic studies have revealed that sugar‐sugar contacts and the C3’‐*endo* conformation of the ribose are also important in i‐motif stability. These factors become particularly significant when chemical modifications are introduced into i‐motif backbone, such as peptide nucleic acids, locked nucleic acids or 2′‐fluorinated residues, showing interesting stabilization properties.^[^ ^18^, ^19^, ^20^, ^21^ ^]^

Among these modifications, the effect of 2′‐modification on i‐motif stability has been specially studied.^[^ ^22^, ^23^ ^]^ Ribocytidine, which adopts a C3’‐*endo* conformation, might be expected to stabilize the i‐motif. However, cytidine‐rich RNA sequences show a low propensity to form i‐motif.^[^ ^24^ ^]^ Thermal stability studies on TC_5_ analogues confirmed that a single rC substitution had a slightly destabilizing effect, whereas double substitution provoked a significant drop in the thermal stability at acidic pH, likely due to steric clashes involving the hydroxyl groups or heavy solvation of these.^[^ ^25^ ^]^

Fluorinated nucleoside analogs have demonstrated an important biological activity as antitumor, antiviral and chemotherapeutic agents.^[^ ^26^, ^27^ ^]^ Fluorination of the sugar moiety alters lipophilicity, electronic density and hydrogen bonding distribution, owing to the small size and highly electronegative nature of the fluorine atom.^[^ ^27^ ^]^ The highly polarized C─F bond, with its strong dipole moment, enhances cell uptake and nuclease resistance, properties that have been exploited in drug design to modulate conformation, pK_a_, intrinsic potency, and pharmacokinetic properties.^[^ ^28^ ^]^

Although fluorine can be considered equivalent to a hydroxyl group, its hydrogen‐bonding capability is weaker.^[^ ^29^ ^]^ Moreover, fluorine provokes significant changes in electronic disposition. The introduction of fluorine at various nucleosides positions can influence conformational preferences through factors such as dipole‐dipole, gauche interactions, antiperiplanar effect or interactions between the F and the base.^[^ ^30^, ^31^ ^]^ For example, 2′‐F analogs exhibit different behaviors. While the 2′‐F in the α‐configuration (2′‐fluororibose) favors the *North* (C3’‐*endo*) conformation, the β form (2′‐fluoroarabino) shows more diverse and less restrictive sugar puckering.^[^ ^32^ ^]^

In i‐motif forming sequences containing 2′F‐araC, i‐motif stability is significantly enhanced across a wide pH range. Structural studies revealed that the derivative adopts a C2’‐*endo* conformation, and that the charge distribution changes due to the electronegative fluorine atoms, which lead to several favorable sequential and inter‐strand electrostatic interactions.^[^ ^19^, ^33^ ^]^ Remarkably, when most of the cytosines in the human telomerase sequence were substituted with 2′F‐araCs, the thermal stability increased by up to 20 ^°^C.^[^ ^34^ ^]^

Gemcitabine (2′‐deoxy‐2′,2′‐difluorocytidine, dFdC) has been approved by the FDA for the treatment of numerous tumors such as pancreatic, nonsmall lung, breast and ovarian cancers.^[^ ^35^ ^]^ This difluorinated nucleoside is metabolized to its corresponding triphosphate nucleoside inhibiting DNA and RNA synthesis and/or ribonuclease reductase activity. Calculations on the molecular structure and electronic properties of gemcitabine were analyzed to provide valuable insights into its interaction with biomolecules.^[^ ^36^ ^]^ In particular, the orientation of dihedral and bond‐bond angles were analyzed by experimental NMR and theoretical DFT studies in several solvents showing different inter‐ and intra‐molecular interactions.^[^ ^37^ ^]^

The synthesis of gemcitabine was originally accomplished by Hertel et al.^[^ ^38^ ^]^ Later, this nucleoside was introduced into synthetic oligonucleotides.^[^ ^39^, ^40^ ^]^ Gemcitabine adopts an RNA‐like C3’‐*endo* conformation that produces structural, electrostatic and thermodynamic changes when introduced in duplexes.^[^ ^41^ ^]^ Several studies in clinical trials have demonstrated the efficacy of the combination of gemcitabine and therapeutic oligonucleotides (antisense oligonucleotides or miRNA) in cancer treatments.^[^ ^42^, ^43^ ^]^ This analog was also introduced into siRNA for gene silencing.^[^ ^44^ ^]^ The results indicated that dFdC had little impact on the structure of siRNA and the inhibition properties were position dependent.

**Scheme 1:**
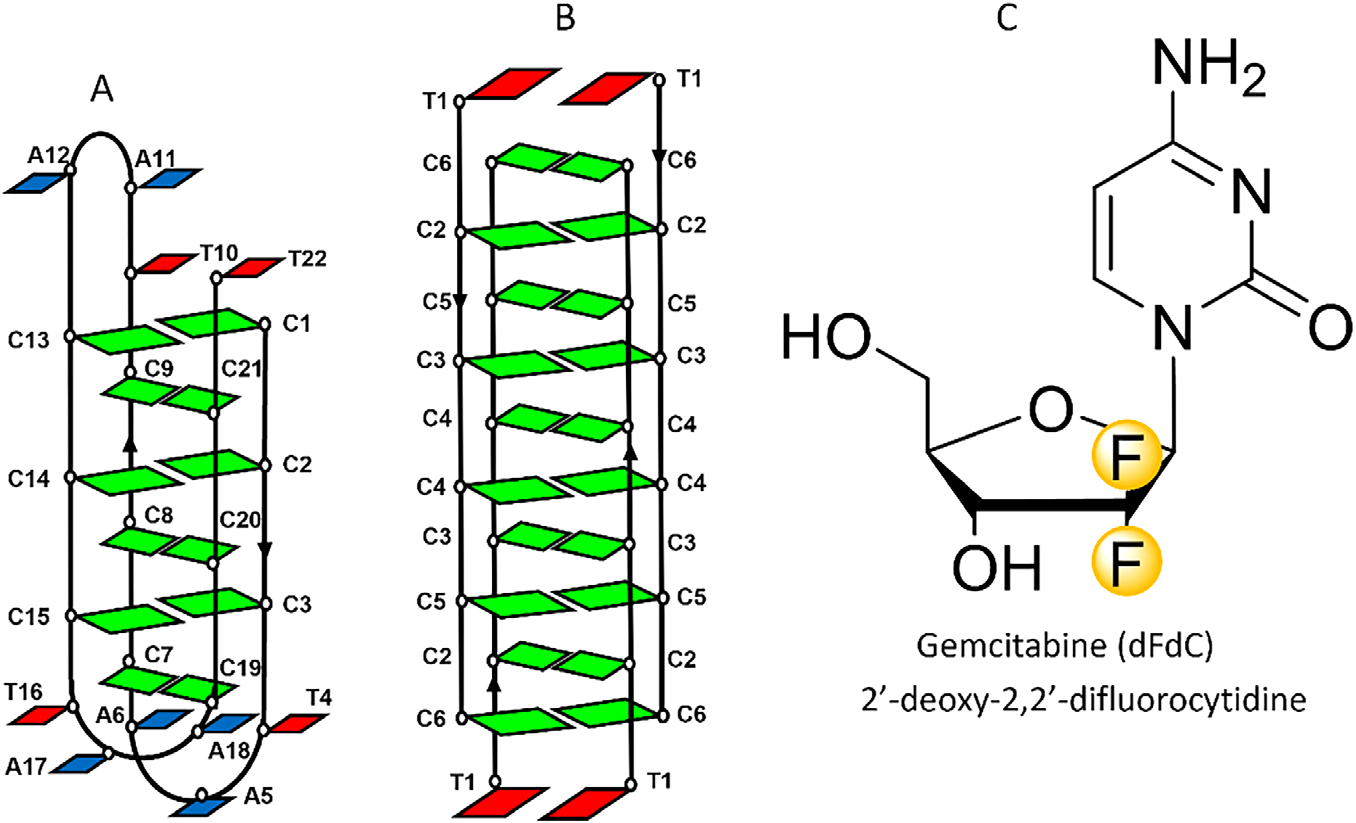
Representation of C‐rich fragment of the tetrameric (TC_5_) i‐motif structure A), the vertebrate telomere (C_3_TA_2_)_3_C_3_T B) and the chemical structure of Gemcitabine (2′‐deoxy‐2′,2′‐difluorocytidine, dFdC). Color code: dC is shown in green and dA and dT in blue and red, respectively.

Inspired by these studies and previous works with single 2′‐fluorinated cytosine analogs,^[^ ^19^, ^21^ ^]^ we investigated the effect of gemcitabine on i‐motif structures. For that, we chose the hexamer TC_5_ i‐motif model that folds into a tetrameric structure containing ten C─C^+^ base pairs,^[^ ^45^ ^]^ and a C‐rich sequence of the vertebrate telomere (C_3_TA_2_)_3_C_3_T, which is known to fold into an intramolecular i‐motif with four cytidine stretches connected by three TAA loops and six C─C^+^ base pairs (Scheme 1).^[^ ^46^ ^]^

**Scheme 2:**
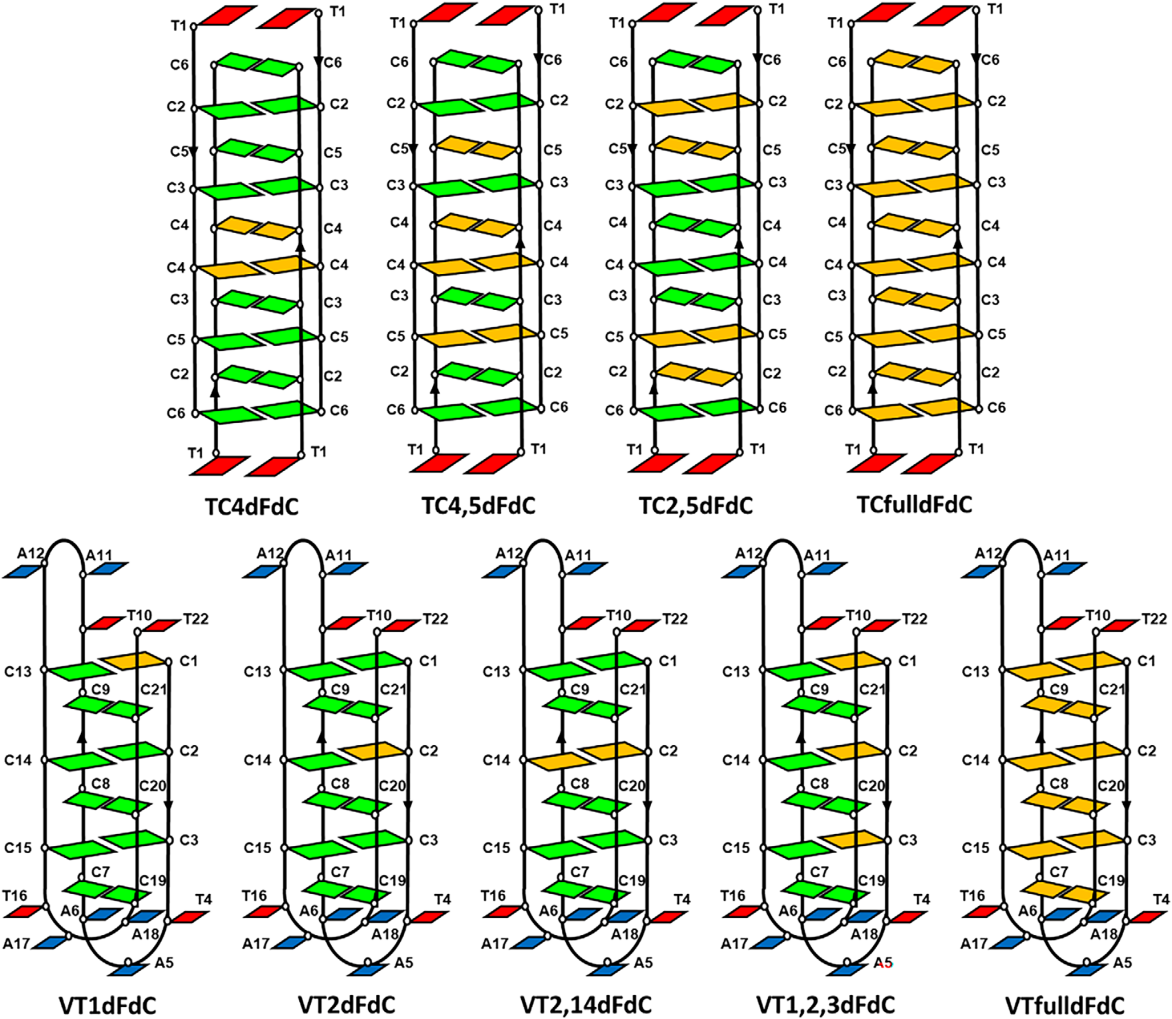
Representation of the VT and TC series of oligonucleotides modified with dFdC. Color code as scheme 1 and dFdC in orange.

Oligodeoxynucleotides (ODN) modified with dFdC in different positions were synthesized to study their effect on the stability and formation of the i‐motif structures. By using a combination of different biophysical and NMR techniques, we observed a clear stabilization of the i‐motif structures when three or more dFdC were located consecutively. Notably, this study presents dFdC as a powerful nucleoside to promote i‐motif structures over a wide range of pH including neutral conditions.

## 2 Experimental Section

### 2.1 Oligonucleotide Synthesis and Purification

Oligonucleotides were synthesized on a 1 µmol scale with an Applied Biosystems 394 synthesizer. All oligonucleotides were synthesized in DMT‐ON mode. After the solid‐phase synthesis, the solid supports were incubated at 55 °C for 6 hours with NH_3_ solution (33 %). The residues were purified using oligonucleotide purification cartridges (Glen Research, USA). All oligonucleotides were analyzed by HPLC, quantified by UV‐absorption at 260 nm and confirmed by MALDI‐TOF mass spectrometry (Table S1).

Gemcitabine phosphoramidite was prepared following the reported methods^[^ ^40^ ^]^ and 2′‐fluoro phosphoramidites, (5′‐Dimethoxytrityl‐N‐acetyl‐2′‐deoxy‐2′‐fluoroarabinocytidine,3′‐[(2‐cyanoethyl)‐(N,N‐diisopropyl)]‐phosphoramidite and 5′‐Dimethoxytrityl‐N‐acetyl‐2′‐deoxy‐2′‐fluorocytidine‐3′‐[(2‐cyanoethyl)‐(N,N‐diisopropyl)]‐phosphoramidite) were obtained from commercial sources (Glen Research, USA).

### 2.2 UV‐Monitored Studies

Absorbance versus temperature curves of i‐motif structures were measured at 4 µM strand concentrations in 20 mM citrate/phosphate (for experiments in which pH ranged from 5.0 to 5.5) or phosphate buffer (for experiments in which pH ranged from 6.0 to 7.0) with 50 mM KCl. Experiments were performed in Teflon‐stoppered 1 cm path‐length quartz cells with a JASCO V‐650 spectrophotometer connected to a Peltier accessory. The samples were heated to 85 °C for 5 minutes, slowly cooled to 25 °C, stabilized at this temperature for a minimum of 5 hours and up to 12 hours, and then stored at 5 °C until the experiments were performed. For the thermal difference spectra (TDS) analysis, UV spectra at low (5 or 15 °C) and high (85 °C) were recorded. The TDS profiles were obtained following described methods.^[^ ^47^ ^]^ Denaturation experiments were performed at 0.5 °C min^−1^ rate to 80 °C, with monitoring the absorbance at 295 and 260 nm. The data were analyzed, and the dissociation temperatures were determined as the midpoint of the transition (T_½_) using in‐house routines written in Matlab (R2009b version; Math‐Works, Natick, MA, USA).^[^ ^48^ ^]^ This analysis assumes a simple two‐state equilibrium between the folded and unfolded states. A self‐complementary tetramolecular process was assumed for thermodynamic analysis of TC_5_ oligonucleotides and an intramolecular process for vertebrate telomer oligonucleotides.

### 2.3 Circular Dichroism (CD) Spectroscopy and Acid‐Base Titrations

Samples previously used for UV denaturing experiments were later analyzed by CD spectrometry. Spectra were registered at 15°C over a range of 220–310 nm, with a scanning speed of 100 nm/min^−1^, a response time of 4 s, a 0.5 nm data pitch and a 1 nm bandwidth using the JASCO spectropolarimeter J‐815 V.

Acid‐base titrations of the VT sequences were monitored by CD following the described conditions. The samples were diluted with 20 mM phosphate buffer with 50 mM KCl and the titrations were carried out by adjusting the pH of solutions containing 2 µM oligonucleotide in a Hellma quartz cell (10 mm path length, 3 mL). CD spectra were recorded in a pH‐stepwise fashion at different pH values. Temperature was set to 20 °C. The data were analyzed following the described methods.^[^ ^49^ ^]^ The pK_a_ of gemcitabine is 3.6.^[^ ^50^ ^]^

### 2.4 Polyacrylamide Gel Electrophoresis (PAGE) Experiments

Samples for the PAGE experiments were prepared at 50 µM oligonucleotide concentration for the VT series and at 100 µM for the TC series in 20 mM phosphate/citrate buffer with 50 mM KCl at pH 5. Before casting, the samples were annealed by increasing the temperature to 85 °C and allowing them to cool down at 10 °C overnight. Samples were supplemented with 12 % glycerol and then loaded in a 20 % nondenaturing gel (19:1 acrylamide: bisacrylamide, Sigma) of 10 × 10 cm at 80 mV for 4.5 hours at 5 °C using 40 mM MES (pH 5) with 1 mM EDTA as a running buffer. After that, the gels were stained with a 0.005 % Stains‐all solution and visualized using an Amersham Imager 680 (GE Life Sciences, Marlborough, MA, USA).

### 2.5 NMR Experiments

Samples for NMR experiments were suspended in 250 µL of either D_2_O or H_2_O/D_2_O 9:1 in 25 mM sodium phosphate buffer. NMR spectra were acquired on Bruker Neo spectrometers operating at 600, or 800 MHz, and processed with Topspin software. TOCSY spectra were recorded with the standard MLEV17 spinlock sequence and with 80 ms mixing time. NOESY spectra in H_2_O were acquired with 75, 150, and 250 ms mixing times. Water suppression was achieved by including a WATERGATE^[^ ^51^ ^]^ module in the pulse sequence prior to acquisition. 2D experiments were carried out at temperatures ranging from 5 to 25 °C. The spectral analysis program Sparky^[^ ^52^ ^]^ was used for semiautomatic assignment of the NOESY cross‐peaks.

### 2.6 Experimental NMR Constraints

Distance constraints were obtained from a qualitative estimation of NOE intensities. NOEs were classified as strong, medium or weak, and distance constraints were set accordingly to 3, 4, or 5 Å. In addition to these experimentally derived constraints, hydrogen bond constraints for the C:C^+^ base pairs were used. Target values for distances and angles related to hydrogen bonds were set to values obtained from crystallographic data in related structures. Force constants were 20 kcal/mol·Å2 for experimental distance constraints, and 30 kcal/mol·Å2 for hydrogen bond distance constraints. Due to the relatively broad linewidths of the sugar proton signals, J‐coupling constants were not accurately measured, but only roughly estimated from DQF‐COSY cross‐peaks. Loose values were set for the sugar dihedral angles δ, ν_1_ and ν_2_ to constrain deoxyribose conformation to *North* or *South* domain. No backbone angle constraints were employed. Distance constraints with their corresponding error bounds were incorporated into the AMBER potential energy by defining a flat‐well potential term.

### 2.7 Structure Determination

Structures were calculated with the SANDER module of the molecular dynamics package AMBER.^[^ ^53^ ^]^ The coordinates of the unmodified telomeric i‐motif (1ELN)^[^ ^46^ ^]^ with the corresponding modifications to include dFdC residues were taken as starting points for the AMBER refinement calculations, consisting of an optimization of the initial coordinates, followed by 1 ns trajectories in which explicit solvent molecules were included and using the Particle Mesh Ewald method to evaluate long‐range electrostatic interactions. The specific protocols for these calculations have been described elsewhere.^[^ ^54^ ^]^ The BSC1 force field^[^ ^54^ ^]^ was used to describe the DNA and the TIP3P model was used to simulate water molecules.^[^ ^55^ ^]^ Force‐field parameters for difluorinated sugars were obtained as described previously.^[^ ^41^ ^]^ Analysis of the representative structures as well as the MD trajectories was carried out with the programs Curves V5.1,^[^ ^56^ ^]^ X3DNA,^[^ ^57^ ^]^ and MOLMOL.^[^ ^58^ ^]^

## 3 Results and Discussion

### 3.1 Design and Synthesis of dFdC‐Modified Sequences

To investigate the impact of gemcitabine modifications on i‐motif structures, a series of C‐rich oligonucleotides were synthesized in which one or more cytosines were replaced by dFdC. Two well‐established i‐motif models were selected: the tetrameric TC_5_ sequence (TC series) and the monomeric human telomeric sequence (VT series). The sequences studied are summarized in Table 1, and their schematic representations, assuming that the modifications do not significantly disrupt the overall folding, are depicted in Scheme 2.

**Table 1:** Sequences synthesized in this study, C corresponds to 2′‐deoxy‐2′,2′‐difluorocytidine, dFdC, C corresponds to 2′‐deoxy‐2′‐fluorocytidine and C corresponds to 2′‐deoxy‐2′‐fluoroarabinocytidine. [Correction added on November 21, 2025, after first online publication: Table 1 has been updated in this version.]

Name	Sequence (5’‐3’)
TCWT	TCCCCC
TC4dFdC	TCCCCC
TC4,5dFdC	TCCCCC
TC2,5dFdC	TCCCCC
TCfulldFdC	TCCCCC
VTWT	CCCTAACCCTAACCCTAACCCT
VT1dFdC	CCCTAACCCTAACCCTAACCCT
VT2dFdC	CCCTAACCCTAACCCTAACCCT
VT2,14dFdC	CCCTAACCCTAACCCTAACCCT
VT1,2,3dFdC	CCCTAACCCTAACCCTAACCCT
VTfulldFdC	CCCTAACCCTAACCCTAACCCT
VT1,2,3FrdC	CCCTAACCCTAACCCTAACCCT
VT1,2,3FadC	CCCTAACCCTAACCCTAACCCT

The TC series represents the simplest i‐motif model, allowing us to examine the effect of hemiprotonated dFdC‐dFdC^+^ pairs. However, the formation of tetrameric structures complicates thermodynamic analysis. Considering that the effect of chemical modification on i‐motifs is frequently nonadditive, the impact of multiple substitutions, both consecutive and nonconsecutive, was explored. **TC4dFdC** is formed by two dFdC‐dFdC^+^ pairs, whereas **TC2,5dFdC** and **TC4,5dFdC** derivatives are formed by four dFdC‐dFdC^+^ pairs. **TCfulldFdC** contains ten dFdC‐dFdC^+^ pairs. In contrast, the VT series permits the study of individual modifications, as single substitutions result in only one change per 3D structure. **VT1dFdC** and **VT2dFdC** are single modified derivatives with one dC‐dFdC^+^ pair, whereas **VT1,2,3dFdC** contains three consecutive modified base pairs. On the other hand, **VT2,14dFdC** and **VTfulldFdC** contain one and six dFdC‐dFdC^+^ pairs, respectively. For comparison, sequences with three consecutive 2′‐deoxy‐2′‐fluorocytidine (**VT1,2,3FrdC**) or 2′‐deoxy‐2′‐fluoroarabinocytidine (**VT1,2,3FadC**) modifications were also prepared.

Oligonucleotides were synthesized and purified using standard phosphoramidite chemistry with N‐benzoyl‐protected dFdC, as previously described.^[^ ^41^, ^59^ ^]^ HPLC and MALDI‐TOF analyses confirmed the identity and purity of all sequences (Table S1).

### 3.2 I‐Motif Formation

**Figure 1:**
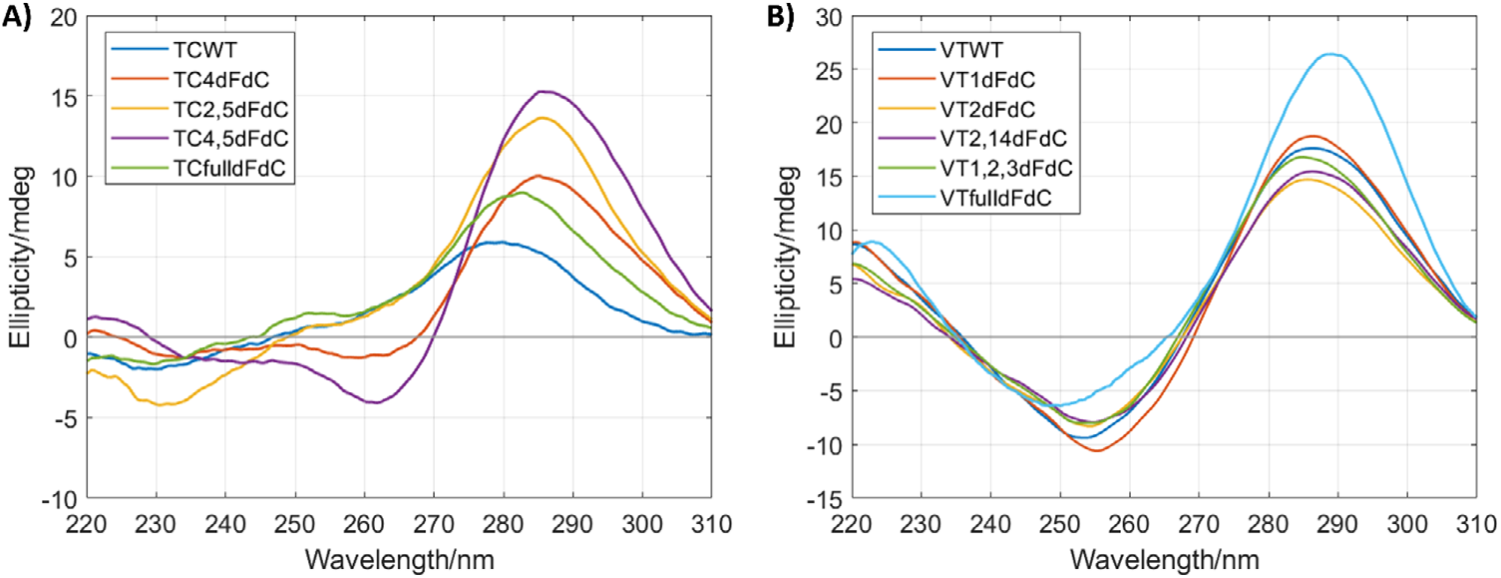
CD spectra of TC A) and VT B) derivatives at pH 6.0. Spectra were recorded at 15 °C in 20 mM phosphate buffer containing 50 mM KCl and 4 µM oligonucleotide concentration.

The formation and stability of i‐motif structures containing dFdC were first assessed by spectroscopic and electrophoretic techniques. CD spectra confirmed i‐motif formation for all derivatives, characterized by a maximum near 290 nm and a minimum at 255 nm. At pH 6, the tetrameric TC series, in which in principle several dFdC‐dFdC^+^ are formed, the i‐motif‐like CD profile was observed for all derivatives except for **TCWT** and **TCfulldFdC** sequences that exhibited broader CD features, consistent with the coexistence of alternative or disordered structures (Figure 1A). At the same pH, VT sequences also displayed the canonical i‐motif profile (Figure 1B), indicating that dFdC‐C^+^ and dFdC‐dFdC^+^ base pairs are well accommodated in this i‐motif assembly. Monofluorinated analogs (2′FriboC and 2′F'araC) exhibited also similar profiles (Figure S1), concluding that di‐ and mono‐fluorinated analogs do not disrupt intramolecular i‐motif folding.

TDS, derived from UV spectra at folded and unfolded temperatures, serve as fingerprints of nucleic acid secondary structures.^[^ ^47^ ^]^ Representative VT and TC sequences were analyzed at pH 6.0 and 7.0 (Figure S2). **VTWT** and **TCWT** exhibited TDS profiles at pH 6 characteristic of i‐motifs, with two positive bands (≈ 240, 265 nm) and one negative band (≈ 295 nm). Interestingly, **VT1,2,3dFdC** and **VTfulldFdC** also showed i‐motif‐like profiles at neutral pH, suggesting enhanced stability conferred by consecutive or multiple dFdC substitutions.

Gel retardation PAGE further validated i‐motif formation at pH 5 (Figure S6). In the VT series, all sequences migrated as single bands, which corresponded to intramolecular i‐motifs. **VTWT, VT1dFdC, and VT2dFdC** displayed light slow‐migrating bands, consistent with a partial unfolding of the structure. For the TC series, single bands were observed for all sequences with similar shifts compared to the T_20_ control, consistent with the tetramerization of the sequences.

^1^H‐NMR spectra revealed signals at 15–16 ppm, characteristic of imino protons in hemiprotonated C:C^+^ base pairs. At pH 5, thymine imino proton signals are observed in the 11.0–11.5 ppm region. **VT1,2,3dFdC,** and **TCfulldFdC** retained C:C^+^ imino signals even at neutral pH.

**Figure 2:**
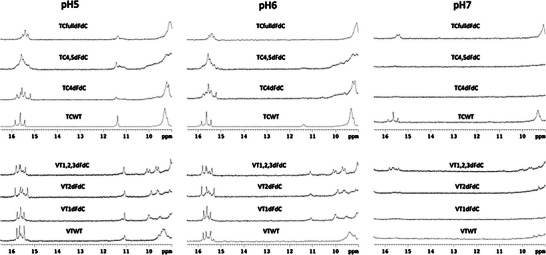
Exchangeable protons regions of the ^1^H NMR spectra of the different modified oligonucleotides at several pH values. Spectra were recorded at 5 °C in 25 mM phosphate buffer and 200 µM oligonucleotide concentration.

Signals in VT derivatives were narrow and well‐dispersed, whereas TC derivatives exhibited broader signals, consistent with the presence of multiple i‐motif species (Figure 2).

**Figure 3:**
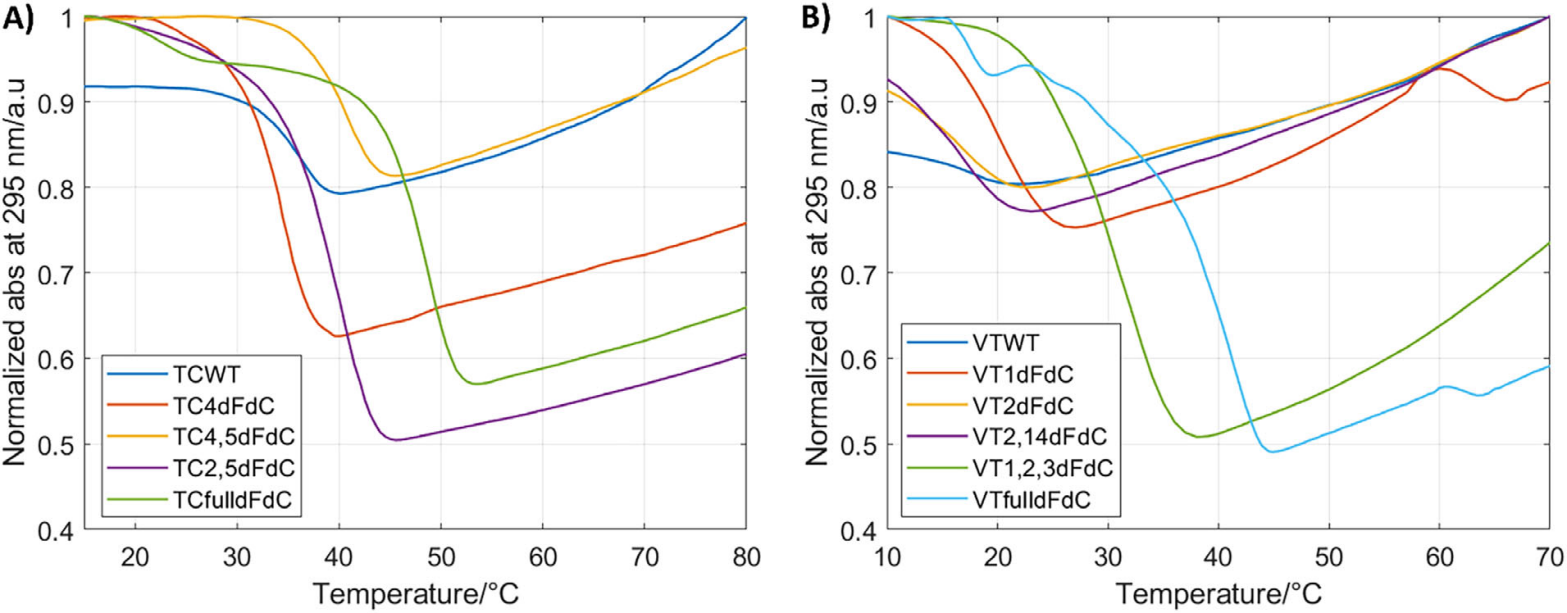
Normalized melting transitions at 295 nm of TC derivatives at pH 6.0 A) and VT derivatives at pH 7.0 B). Melting experiments were carried out from 10 °C (pH 7) or 15 °C (pH 6) up to 80 °C in 20 mM phosphate buffer containing 50 mM KCl, with an oligonucleotide concentration of 4 µM.

### 3.3 Thermal Stability

Thermal denaturation curves were monitored at a pH range of 5.0–7.0 and at 295 and 260 nm. Given the slow kinetics of i‐motif folding/unfolding,^[^ ^10^ ^]^ the apparent melting temperatures (T_½_) and pH midpoints (pH_½_) were recorded. At pH 5.0, single and double dFdC substitutions moderately increased T_½_ (Tables 2 and S2 and Figures 3 and S3). In the VT series, **VT1dFdC** showed stabilization at other pH values, whereas **VT2dFdC** and **VT2,14dFdC** were destabilized above pH 5.0. In the TC series, a single substitution (**TC4dFdC**) had a minimal effect, while multiple substitutions were stabilizing at all pH values. Remarkably, the tri‐substituted **VT1,2,3dFdC** exhibited substantial stabilization (∆T_½_ = 10.2 °C at pH 6.5 and 18.6 °C at pH 5.0). **VTfulldFdC** showed even higher stabilization (∆T_½_ = 22.2 °C at pH 7.0 and > 30 °C at lower pH), with an additional transition at ≈ 10 °C, suggesting alternative species. Fully substituted TC sequences displayed enhanced stability at acidic pH. Conversely, this effect was not observed at a neutral pH. Multiple transitions in TC sequences likely correspond to alternative i‐motif arrangements or higher‐order structures. Transitions monitored at 260 nm followed the same trend as those at 295 nm (Table S2).

**Table 2:** Apparent T_½_ values^[^ ^a^ ^]^ in °C for TC and VT oligonucleotides at 295 nm. The uncertainty is ± 1 °C. Buffer conditions: 20 mM phosphate/citrate (pH 5) or phosphate (pH 6 and pH 7), 50 mM KCl, strand concentration 4 µM.

Name	T_½_ pH 7.0	T_½_ pH 6.5	T_½_ pH 6.0	T_½_ pH 5.5	T_½_ pH 5.0
TCWT	n.d^[^ ^b^ ^]^	27.8	36.8	41.9	48.3
TC4dFdC	n.d	n.d	36.4 (‐0.4)^[^ ^c^ ^]^	40.5 (‐1.4)	48.9 (+0.6)
TC4,5dFdC	n.d	30.0 (+2.2)	39.6 (+2.8)	44.1 (+2.2), 17.9	52.9 (+4.6)
TC2,5dFdC	n.d	n.d	36.0 (+0.8)	48.3 (+6.4)	51.0 (+2.7)
TCfulldFdC	n.d	29.8 (+2.0)	51.1 (+14.3), 22.0	58.2 (+16.3), 17.2	67.5 (+19.2), 27.8
VTWT	17.9	20.8	27.5	41.3	47.3
VT1dFdC	21.5 (+3.6)	23.0 (+2.2)	29.2 (+1.7)	41.5 (+0.2)	51.0 (+3.7)
VT2dFdC	16.5 (‐1.4)	21.0 (+0.2)	26.7 (+0.1)	40.1 (‐1.4)	49.2 (+1.9)
VT2,14dFdC	16.4 (‐1.5)	19.7 (‐1.1)	25.9 (‐1.6)	41.4 (+0.1)	50.1 (+2.8)
VT1,2,3dFdC	30.0 (+12.1)	31.0 (+10.2)	40.6 (+13.1)	54.8 (+13.5)	65.9 (+18.6)
VTfulldFdC	40.1 (+22.2)	51.1 (+30.3), 41.1	64.7 (+37.2), 52.7	76.4 (+35.1), 60.6	>80

^[a]^ Apparent Tm values are reported due to presence of multiple species in the folded states.

^[b]^ n.d: T_½_ is not determined because the profile of the melting transitions is broad.

^[c]^ T_½_ increments related to TCWT and VTWT are shown between brackets.

NMR melting studies at pH 5 and 6 corroborated the UV data. Most derivatives retained imino signals above 35 °C, except for **VTWT** and **VT1dFdC** at pH 6. **VT1,2,3dFdC** and **VTfulldFdC** exhibited highly stable, sharp, and well‐dispersed signals even at neutral pH. TC derivatives showed broader and less dispersed imino signals, consistent with the formation of multiple i‐motif species.

Based on our results that the proximity of multiple dFdC modifications seemed to increase the stability of the overall i‐motif structure, we wanted to explore if this effect was exclusive of dFdC or was also attributable to monofluorinated derivatives. To gain further insights, **VT1,2,3FadC** and **VT1,2,3FrdC** oligonucleotides with three consecutive monofluorinated modifications were also prepared.

We observed, in accordance with previous CD experiments, that the monofluorinated modifications also contribute to the stabilization of this particular i‐motif structure across pH values, although the stabilities observed were not as significant as those observed with dFdC (Table S3).

### 3.4 Circular Dichroism Monitored Acid‐Base Experiments

Acid‐base titrations of the VT sequences were performed by recording the CD spectra at different pH conditions. Figure S4 illustrates the titration profiles of the different VT sequences by displaying the fraction of folded (protonated) structures as a function of pH. **VT1,2,3dFdC** exhibited a remarkable stabilizing effect at neutral pH compared to the mono‐ and di‐modified derivatives. The pH_½_ values for mono‐ and di‐substituted derivatives are quite similar to the unmodified one (pH_½_ = 6.53), whereas the pH_½_ of the tri‐substituted derivative is significantly shifted to 7.1. **VTfulldFdC** exhibited a different pH‐titration profile, characterized by two transitions that were analysed at 280 nm. In addition, this experiment was analysed by a multivariate method using a model of three species.^[^ ^60^, ^61^ ^]^ The pH transition midpoints, pH_½_ are 6.76 and 7.34 being the second transition strongly cooperative. Table 3 provides the pH_½_ and transition ranges obtained from the titration data.

**Table 3:** pH transition midpoints and transitional pH range for VT sequences determined from CD‐monitored acid‐base titrations. Titrations performed in 20 mM phosphate buffer containing 50 mM KCl, with an oligonucleotide concentration of 2 µM.

Name	pH_½_	pH 10%	pH 90%	Transitional pH range
VTWT	6.53 ± 0.08	6.18 ± 0.08	6.87 ± 0.08	0.69 ± 0.08
VT1dFdC	6.59 ± 0.06	6.26 ± 0.06	6.91 ± 0.06	0.65 ± 0.06
VT2dFdC	6.51 ± 0.07	6.10 ± 0.07	6.92 ± 0.07	0.82 ± 0.07
VT2,14dFdC	6.54 ± 0.04	6.33 ± 0.04	6.75 ± 0.04	0.42 ± 0.04
VT1,2,3dFdC	7.08 ± 0.06	6.60 ± 0.06	7.57 ± 0.06	0.97 ± 0.06
VTfulldFdC	6.76 ± 0.09, 7.34 ± 0.07	6.15 ± 0.08	7.70 ± 0.08	1.55 ± 0.08

TC derivatives also showed the expected i‐motif CD profiles at acidic pH (5.0–5.5), with some derivatives maintaining i‐motif features at pH 6 (Figure S5). Fully substituted **TCfulldFdC** exhibited typical i‐motif spectra at low pH, although alternative strand‐sliding structures cannot be excluded.

### 3.5 Structural Analysis by NMR

**VT1,2,3dFdC** was selected for detailed NMR analysis due to its high stability and well‐dispersed spectra. **VTfulldFdC** spectra were also of good quality, but extensive substitutions, lacking the key H2'/H2‘’ protons, complicated the assignment of key NOE contacts.

**Figure 4:**
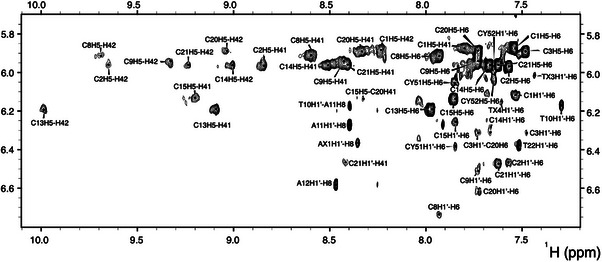
Region of the NOESY spectra of VT1,2,3dFdC showing H1’/aromatic and amino/aromatic regions (mixing time 250 ms, T = 5 °C, pH 5.0). Spectrum performed in 25 mM phosphate buffer, with an oligonucleotide concentration of 500 µM.

Cytosine and dFdC resonances were identified via H5–H6 TOCSY cross‐peaks and H5–amino NOEs. Sugar–base connectivity was established through H6–H1′ NOEs, and dFdC residues were distinguished by characteristic H1′ splitting due to ^1^H–^1^⁹F couplings. Hemiprotonated base pairs were mapped via sequential NOE pathways. 2D NOESY spectra revealed all characteristic NOE patterns of i‐motif structures (Figure 4 and S9, S10). Minor groove H1′–H1′ NOEs (Figure S10), together with sequential contacts, allowed unambiguous assignment of central core residues. The data indicate a structure highly similar to the unmodified **VTWT**.

**Figure 5:**
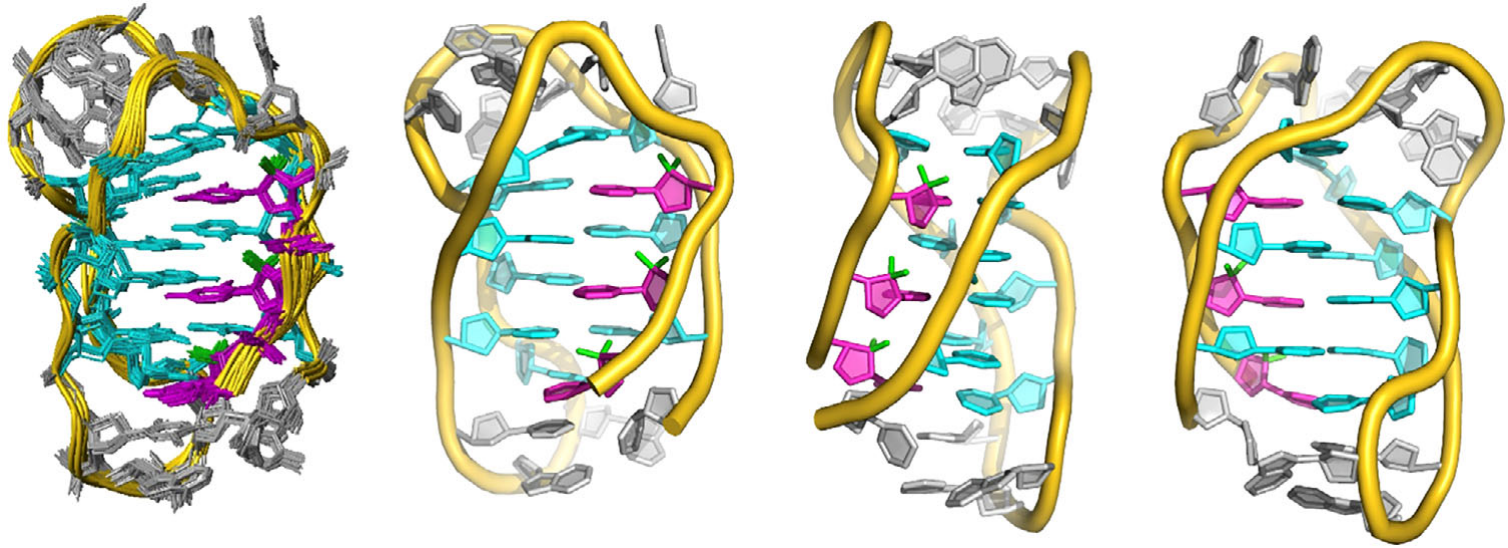
Ensemble of the 10 determined structures of VT1,2,3dFdC, and several views of the average structure. Color code: dC in cyan; dFdC in magenta; and dA and dT loop residues in grey. Fluorine atoms are shown in green.

Although the number of experimental ^1^H‐^1^H distance constraints is limited by the extensive fluorine substitutions, a structural model of VT1,2,3dFdC was built using the structure of the human telomeric C‐rich strand (PDB 1ELN) as a template and refined by restrained molecular dynamics with AMBER, incorporating experimental distance constraints. The final model is shown in Figure 5.

## 4 Discussion

Chemical modifications in i‐motifs are highly relevant for a variety of biotechnological applications. Among these, (2′F‐araC) and (2′F‐riboC) have received particular attention due to their potential in antisense oligonucleotides and small interfering RNA (siRNA) technologies. These modifications are resistant to nuclease degradation and compatible with in vivo applications, making them attractive candidates for therapeutic oligonucleotides. Importantly, both 2′F‐araC and 2′F‐riboC have been shown to be compatible with the model TC_5_ i‐motif formation, although their effects on structural stability differ substantially. While 2′F‐araC strongly stabilizes the i‐motif relative to unmodified DNA, 2′F‐riboC has been observed to exhibit minimal stabilizing effect.^[^ ^21^, ^23^ ^]^ The present study explores the impact of gemcitabine (dFdC) on i‐motif stability, representing a logical extension of previous work on monosubstituted fluorinated nucleosides.

The incorporation of dFdC into nucleic acids has been less studied compared to 2′‐monofluoro derivatives. Previous work in DNA:RNA hybrids and self‐complementary DNA duplexes indicated that dFdC generally induces only minor destabilization and slight structural distortion.^[^ ^41^ ^]^ Sugar conformation analysis revealed that dFdC adopts a *North*‐type (C3’‐*endo*) sugar pucker in B‐type DNA duplexes and a *South*‐type (C2’‐*endo*) conformation in DNA:RNA hybrids (A‐type duplexes). Notably, one of the 2′‐fluorine atoms was observed to interact with the 3′‐neighboring purines, highlighting the significant influence of fluorine atoms on base pairing interactions. These observations suggested that dFdC is a highly adaptable modification capable of accommodating different structural contexts.

**Figure 6:**
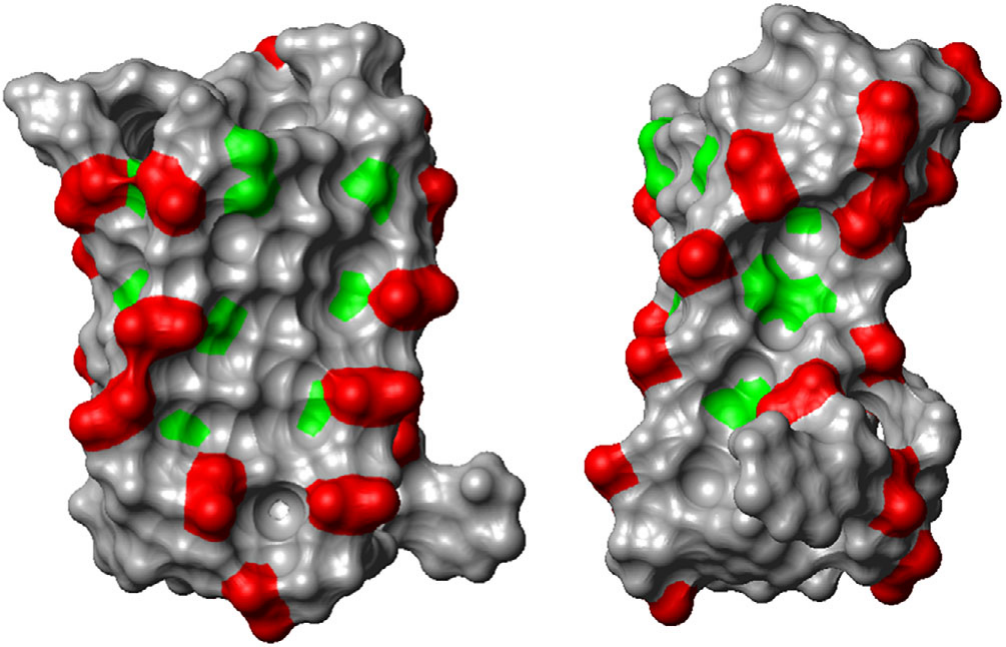
Two views from major and minor grooves, respectively, of the molecular surface of the model structure of VTfulldFdC. Surface regions close to phosphate groups are shown in red and close to fluorine atoms in green.

The present study demonstrates that dFdC exerts a more pronounced effect in the context of intramolecular and tetramolecular i‐motifs than in canonical double‐helical structures. Our results suggest that both dFdC:C^+^ and dFdC:dFdC^+^ base pairs provide comparable stability to unmodified C:C^+^ base pairs, and that i‐motif folding is maintained across all examined derivatives. Remarkably, **VT1,2,3dFdC**, which contains three consecutive dFdC residues, and particularly **VTfulldFdC**, exhibit exceptional thermal stability even at neutral pH. Furthermore, these sequences also display a significant shift in the pH_½_ of folding toward physiological conditions, indicating a substantial stabilization effect. The results suggest that a “fluorine spine” along the i‐motif grooves, formed by either a single track of three consecutive dFdC residues (**VT1,2,3dFdC**) or four tracks in **VTfulldFdC**, contributes strongly to i‐motif stabilization. The model of **VTfulldFdC** (Figure 6) illustrates how the fluorine atoms remain solvent‐accessible along both the major and minor grooves, suggesting that changes in the hydration of these fluorine‐rich patches may further influence overall stability. Although the presence of multiple species complicates the analysis in tetrameric TC sequences, the trends observed are consistent with the monomeric VT series, reinforcing the generality of these findings.

Previous studies involving 2′F‐araC and 2′F‐riboC suggest that the effect of fluorine on i‐motif stability arises from two opposing contributions. Fluorine in the β position can form favorable interactions with positively charged groups in the major groove, thereby stabilizing the structure, as observed in iFANA‐modified i‐motifs.^[^ ^19^, ^33^ ^]^ These studies show that 2′F‐araC‐dC^+^ and 2′F‐araC‐2′FaraC^+^ base pairs are compatible with i‐motif formation. In contrast, fluorine in the α position has a different behavior, while in intramolecular i‐motif can be accommodated by forming 2′F‐riboC‐dC^+^ base pairs, as observed in this work. Previous results have shown that, when introduced in tetramolecular TC i‐motifs, this modification may disrupt sugar‐sugar contacts in the minor groove, destabilizing the structure.^[^ ^21^, ^23^ ^]^ In contrast, our results suggest that the adaptability of dFdC's sugar conformation likely mitigates these unfavorable interactions.

Gemcitabine is unique among nucleosides in containing two fluorine atoms at the 2′‐position. The high electronegativity of fluorine polarizes the C─F bond, producing a low‐lying C─F σ* orbital capable of hyperconjugative interactions. These effects enhance dipole, electrostatic, and hydrogen bonding interactions, influencing oligonucleotide conformation. We hypothesize that dFdC residues in i‐motifs, may align according to the “gauche effect,” whereby the C─F σ* orbital of one dFdC accepts electron density from adjacent C─H σ bonds. This type of interactions, sometimes referred to as pseudohydrogen bonds, have been observed by NMR and characterized through quantum mechanical (QM) calculations in other nucleic acids containing 2′F‐ANA derivatives.^[^ ^62^, ^63^ ^]^ Classical hydrogen bonds, such as C─F···H─O and C─F···H─N interactions, have also been reported in 2′F‐ANA‐modified i‐motifs.^[^ ^33^ ^]^ Although our model does not reveal contacts close enough to conclusively confirm these interactions, its precision is insufficient to rule out their occurrence—particularly when multiple dFdC residues are in close proximity and under acidic conditions, which favor hydrogen‐bonding networks between deoxyribose sugars in the four‐stranded core.

Interestingly, while the stabilizing effect of 2′F‐araC is largely additive,^[^ ^33^ ^]^ the destabilizing effect of 2′F‐riboC is in some cases less so,^[^ ^21^ ^]^ which may help explain the strong cooperative stabilization observed for multiple consecutive dFdC substitutions. However, this effect alone is unlikely to fully account for the extraordinary stability of sequences such as **VTfulldFdC**. Additional factors, distinct from those operating in monosubstituted derivatives, are also likely to contribute. For example, in double‐helical RNA, stabilization of 2′F‐RNA relative to RNA has been attributed to fluorine‐induced changes in hydration and nucleobase polarization. Dehydration effects may play a key role in i‐motif stabilization as well, particularly in the light of evidence that i‐motifs are stabilized under crowding or dehydrating conditions.^[^ ^64^, ^65^ ^]^ The cooperative effects observed with multiple consecutive dFdC substitutions may therefore reflect both local hyperconjugative interactions and global effects mediated by changes in hydration and backbone polarization.

Finally, it is noteworthy that the cooperative stabilization effect of dFdC becomes significantly more pronounced under acidic conditions. This property opens the way to its potential use in applications where thermal stabilization of nucleic acid structures at defined pH values is needed, such as the selective stabilization of nucleic acid assemblies within acidic cellular compartments. Moreover, since gemcitabine has been extensively used as a chemotherapeutic agent, its incorporation into oncogenic and telomeric regions could potentially lead to the stabilization of i‐motif structures—which have been associated with the regulation of gene expression, replication, and genome stability—thereby expanding its possible mechanisms of action, as reported for other cytidine analogues.^[^ ^66^ ^]^

## 5 Conclusion

In conclusion, dFdC represents a powerful modification for stabilizing i‐motif structures. The remarkable thermal and pH stability of the compound, which exceeds that of previous monosubstituted fluoronucleosides, is attributable to its dual fluorine atoms, adaptable sugar conformation, and capacity for cooperative interactions when multiple consecutive dFdC residues are introduced. These findings open new avenues for the design of chemically modified i‐motifs with enhanced stability under physiological conditions, with potential applications in nanotechnology, therapeutic oligonucleotides, and pH‐responsive biomolecular devices.

## Conflict of Interest

The authors declare no conflict of interest.

## Supporting information



Supporting Information

## Data Availability

The data that support the findings of this study are available on request from the corresponding author. The data are not publicly available due to privacy or ethical restrictions.
